# Parental alcohol consumption during pregnancy and mental health of their children up to adulthood

**DOI:** 10.1192/j.eurpsy.2025.661

**Published:** 2025-08-26

**Authors:** Z. Mohrova, Z. Csajbók, L. Andryskova, A. Ksinan, P. Brennan Kearns

**Affiliations:** 1 Second Faculty of Medicine; 2 Faculty of Humanities, Charles University, Prague; 3 RECETOX, Faculty of Science, Masaryk University, Brno, Czech Republic

## Abstract

**Introduction:**

Alcohol consumption of mothers can lead to problems in emotional and behavioural development of children. However, less is known about the effects of paternal alcohol drinking.

**Objectives:**

We aimed to investigate whether maternal or paternal alcohol consumption during pregnancy longitudinally affected children’s mental health.

**Methods:**

We analyzed a total of 2,013 parent-child triads (52% of children were males) from the European Longitudinal Study of Pregnancy and Childhood. Data on alcohol consumption was obtained from questionnaires from both parents during pregnancy and after the child’s birth. Mental health and behaviour of children was assessed with Strength and Difficulties Questionnaire (SDQ) at ages 7, 11, 15, and 18 years old, as reported by mothers and children themselves. The associations were tested using linear regression, adjusting for parent’s age at child’s birth, child’s sex, and other socio-demographic and psychosocial covariates. We also tested an interaction between the exposure and children’s sex.

**Results:**

Maternal alcohol consumption was associated with higher total SDQ scores at ages 7, 11, and 18 years old when the outcomes were reported by mothers, but only at 11 when reported by children. We did not observe any dose-response relationship, and the effect size did not change during the follow-up. Results of the linear regressions are displayed in Table 1. We did not detect any effect modification by child’s sex. The effects were observed across various domains of SDQ (except for the peer problems subscale): in the emotional symptoms subscale at age 11 when reported by both mother and child, in the conduct problems subscale at ages 7 and 11 when reported only by mother, in the hyperactivity/inattention subscale at age 18 when reported only by mother. Paternal alcohol consumption was not associated with the total SDQ score.

**Table 1:** Association of maternal alcohol consumption with the total score of Strength and Difficulties Questionnaire

		Maternal alcohol consumption during pregnancy	
		Once	Twice or thrice
	Child’s age (years)	B (95% confidence interval)	
SDQ reported by mothers	7	0.4 (-0.1, 0.9)	0.7 (0.1, 1.3)*
	11	0.7 (0.2, 1.2)*	0.6 (0.1, 1.2)*
	15	0.6 (0.0, 1.3)	0.5 (-0.2, 1.2)
	18	0.8 (0.0, 1.6)*	0.9 (0.1, 1.7)*
SDQ reported by children	11	0.1 (-0.4, 0.7)	0.8 (0.2, 1.5)*
	15	0.4 (-0.5, 1.2)	0.5 (-0.4, 1.4)
	18	0.1 (-1.4, 1.5)	-0.1 (-1.4, 1.2)

* p-value < .05. The reference category is no alcohol consumption. Results are from a fully adjusted model.

**Conclusions:**

Maternal alcohol consumption has a long-term effect on children’s mental health in particular when reported by mothers. Interventions preventing maternal alcohol consumption during pregnancy may protect children’s mental health.

**Disclosure of Interest:**

None Declared

